# In situ relief of postrepair pulmonary venous obstruction using the endocardial anchoring technique

**DOI:** 10.1016/j.xjtc.2024.04.009

**Published:** 2024-04-27

**Authors:** Yusuke Yamamoto, Sho Akiyama, Kentaro Hotoda, Mio Noma, Shuhei Yamaguchi, Hiroki Nagamine, Jun Maeda, Yukihiro Yoshimura

**Affiliations:** a Department of Pediatric Cardiovascular Surgery, Tokyo Metropolitan Children's Medical Center, Tokyo, Japan; b Department of Pediatric Cardiology, Tokyo Metropolitan Children's Medical Center, Tokyo, Japan

**Figure:**
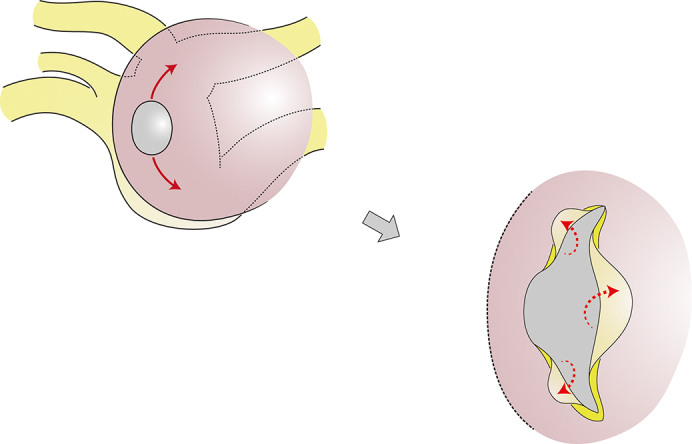
The endocardial anchoring technique for isolated anastomotic stenosis after TAPVR repair.

**Central Message**

The endocardial anchoring technique is a feasible maneuver for in situ relief of isolated anastomotic stenosis after TAPVR repair, providing a wide and smooth orifice with growth potential.

Outcomes of the conventional repair for total anomalous pulmonary venous return (TAPVR) remain suboptimal mainly due to the occurrence of progressive pulmonary venous (PV) obstruction. In general, these postrepair PV obstructions are treated with the sutureless technique, although direct enlargement of the stenotic junction might be an alternative in those without obstruction of the peripheral PVs. Herein, we describe a patient with isolated anastomotic stenosis after conventional repair of infracardiac TAPVR that was successfully managed using in situ repair by the endocardial anchoring technique. This novel maneuver includes drawing the incised wall of the PV confluence into the left atrium and anchoring it to the atrial endocardium.^1^^,^^2^ Both parents of the patient provided written informed consent for publication of this report (institutional review board number and date: 2023b-170; March 11, 2024).

## Case Presentation

**Figure E1:**
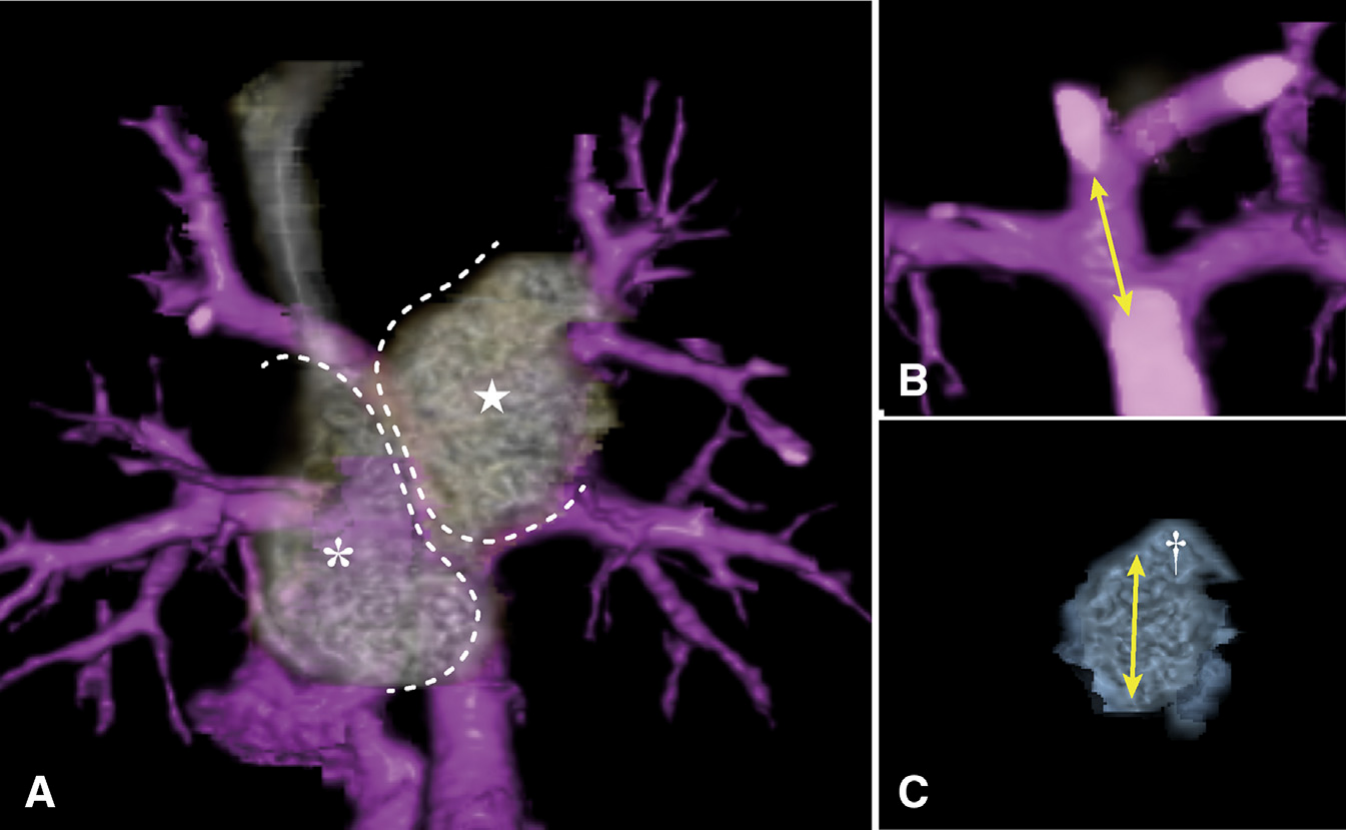
Volume rendering images of computed tomography (*CT*) angiography taken before the primary repair of total anomalous pulmonary venous return. A, The entire image of the pulmonary venous (*PV*) system viewed from the front, with the posterior wall of the left atrium drawn in transparent *pale blue*. The *outlines* of *left* (*star*) and right atrium (*asterisk*) are indicated by *white dotted lines*. B and C, Incision lines on both the PV confluence and the left atrium in the primary operation are indicated by *yellow double headed arrows*, wherein 2 lines are arranged in a slightly twisted position because of anatomical limitations. Left atrial appendage is indicated by a *white dagger*.

The patient was born at 38 weeks’ gestation with a birth weight of 2.8 kg, and was diagnosed with infracardiac TAPVR. On postnatal day 1, direct anastomosis between the left atrium and PV confluence was performed, along with ligation of both the ductus arteriosus and the vertical vein, and patch closure of the atrial septal defect (Figure E1). Although the postoperative course was uneventful with normalized hemodynamic parameters, stenosis of the anastomosis site was identified at 6 months postoperatively. During close follow-up, the mean pressure gradient across the junction gradually increased, finally reaching 7 mm Hg at age 1 year and a body weight 8 kg. Hence, surgical relief of the PV obstruction was indicated.

## Technique

**Figure 1:**
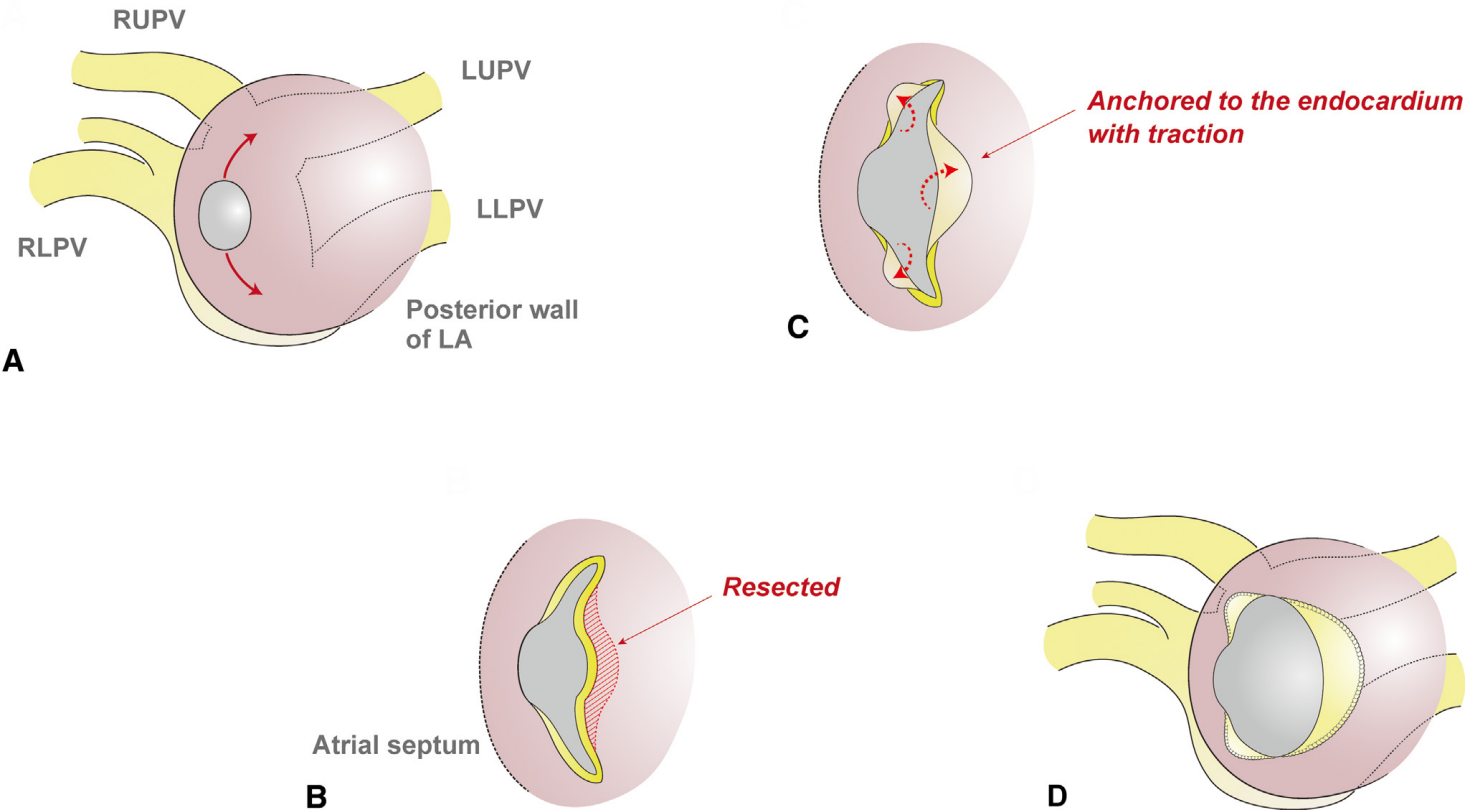
Schematic view of the endocardial anchoring technique. A, The posterior wall of the left atrium and the adjacent anterior wall of the pulmonary vein (*PV*) confluence were collectively incised upward, and then leftward. B, The redundant part of the left atrial wall was resected to not obstruct the subsequent transfer of the flap of the PV confluence. C, The 3 components of the free edge of the incised PV confluence were respectively drawn into the left atrial cavity. D, The flaps were anchored to the endocardium of the left atrium with a running suture, applying an adequate traction force onto the reversed flap.

**Figure 2:**
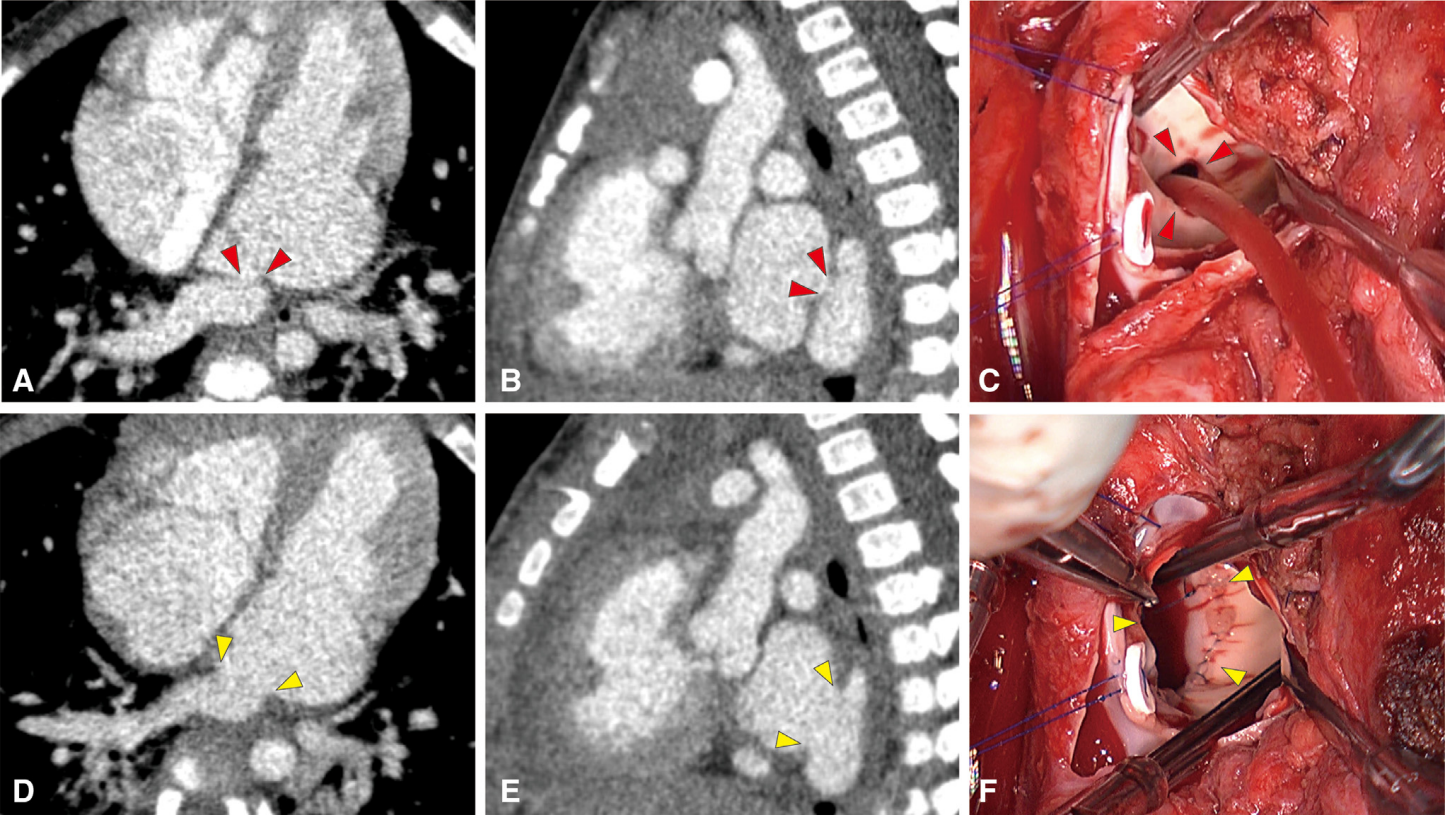
Pre- and postrepair images. A and B, Pre-repair computed tomography (*CT*) images showing the stenotic orifice (*red arrowheads*). C, Intraoperative image showing the stenotic orifice through the incised interatrial septum (*red arrowheads*). D and E, Postrepair CT images showed a widely opened orifice (*yellow arrowheads*). F, Intraoperative picture showing the enlarged orifice (*yellow arrowheads*).

After cardioplegic cardiac arrest, the anastomotic site, which was a tiny hole 6 mm in diameter, was exposed through a longitudinal incision of the interatrial septum (Video 1). The posterior wall of the left atrium and the adjacent anterior wall of the PV confluence were collectively incised upward and leftward, taking care not to extend it to the individual PVs (Figure 1). Then, 3 components of the free edge of the incised PV confluence were respectively drawn into the left atrial cavity. These flaps were anchored to the endocardium of the left atrium with a running suture, applying an adequate traction force onto the reversed flaps. To fully mobilize the flap of the PV confluence, additional dissection between the posterior wall of the left atrium through the cut edge of the incision, followed by resection of the obstructive atrial wall. Postoperative computed tomography imaging showed a widely opened connection between the PV confluence and the left atrium (Figure 2). Follow-up echocardiography at half a year postoperatively also revealed laminar flow with minimal pressure gradient at the enlarged connection (Video 2).

## Discussion

Currently, postrepair PV obstruction is usually treated by the sutureless technique with satisfactory outcomes.^3^ Nevertheless, for patients with isolated anastomotic stenosis, direct enlargement of the stenotic junction should be considered as a simple, less-invasive alternative. Because this novel technique is feasible through a transseptal approach, thorough dissection of the retrocardiac space is not necessarily required, allowing surgeons to avoid postoperative complications such as hemorrhage and phrenic nerve injury. Theoretically, the seamless configuration created by this technique is supposed to prevent turbulent blood flow and subsequent intimal hyperplasia, in contrast to the nonphysiological shape of the channel for pulmonary venous drainage after repair by the sutureless technique. In addition, the traction force generated by anchoring of the flap might allow preservation of the patency of the orifice, protecting against recurrence of the anastomotic stenosis. On the other hand, circumferential suture line could be disadvantageous in regard to the growth potential of the reconstructed orifice. To address this concern, we divide the suture line into 3 parts while preserving the continuity of native tissue along approximately one-quarter of the orifice. Further follow-up is necessary to reveal the long-term outcome of this technique. In addition, fluid dynamic study of the PV drainage channel, in comparison with the sutureless repair, remains a subject for future investigation.

## Conflict of Interest Statement

The authors reported no conflicts of interest.

The *Journal* policy requires editors and reviewers to disclose conflicts of interest and to decline handling manuscripts for which they may have a conflict of interest. The editors and reviewers of this article have no conflicts of interest.

## Supplementary Data

Video 1 The endocardial anchoring technique for relief of isolated anastomotic stenosis after the conventional repair of infracardiac total anomalous pulmonary venous return. Video available at: https://www.jtcvs.org/article/S2666-2507(24)00167-6/fulltext.

Video 2 Postoperative computed tomography imaging and echocardiogram. Video available at: https://www.jtcvs.org/article/S2666-2507(24)00167-6/fulltext.
