# Supplementary figures and images for: In situ relief of postrepair pulmonary venous obstruction using the endocardial anchoring technique
Source: JTCVS Tech. 2024 Apr 27;26:96–9. doi: 10.1016/j.xjtc.2024.04.009 (PMC11329171; doi:10.1016/j.xjtc.2024.04.009)

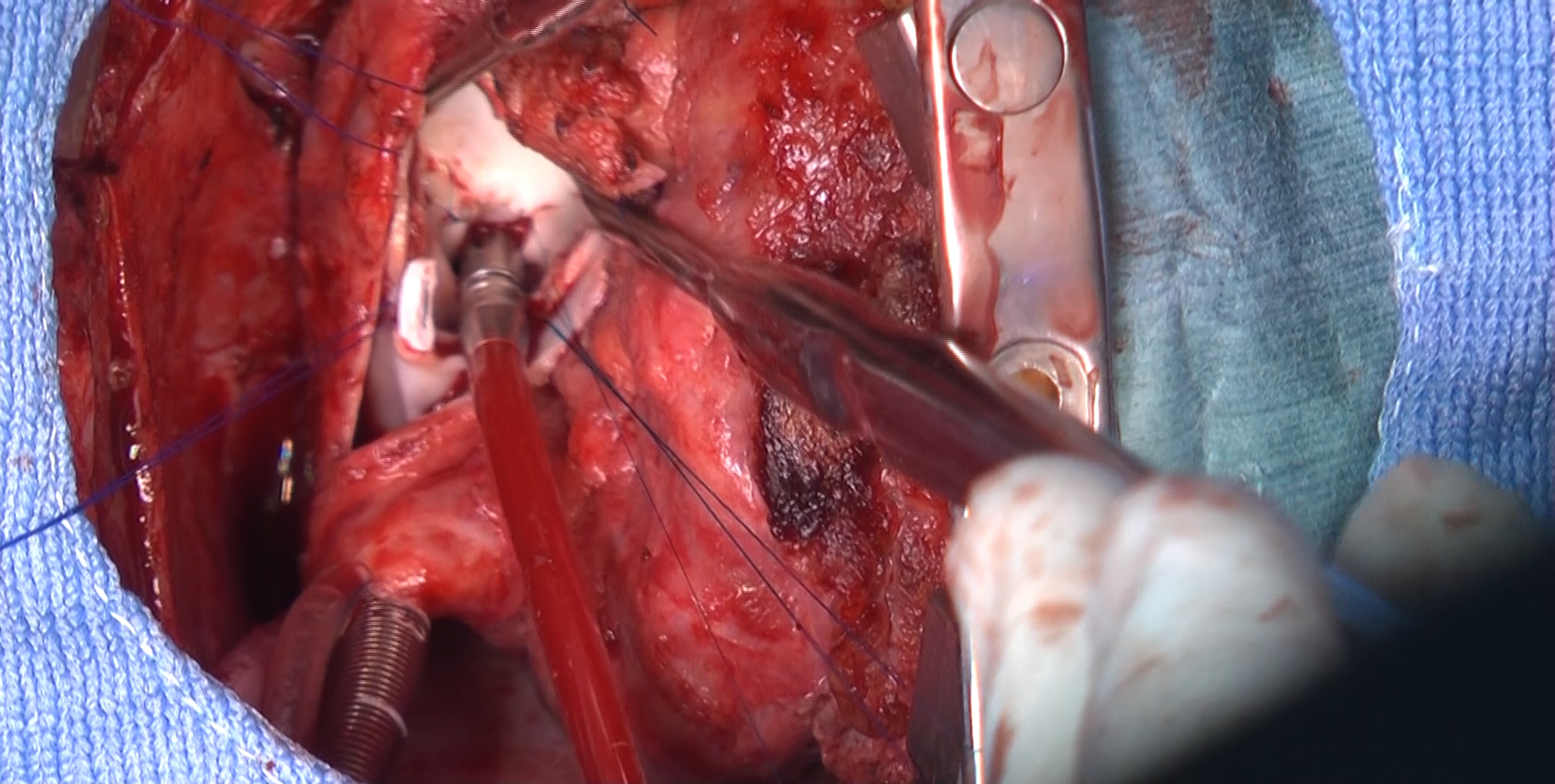

Supplement: Video 1 — The endocardial anchoring technique for relief of isolated anastomotic stenosis after the conventional repair of infracardiac total anomalous pulmonary venous return. Video available at: https://www.jtcvs.org/article/S2666-2507(24)00167-6/fulltext. [file fx2.jpg]

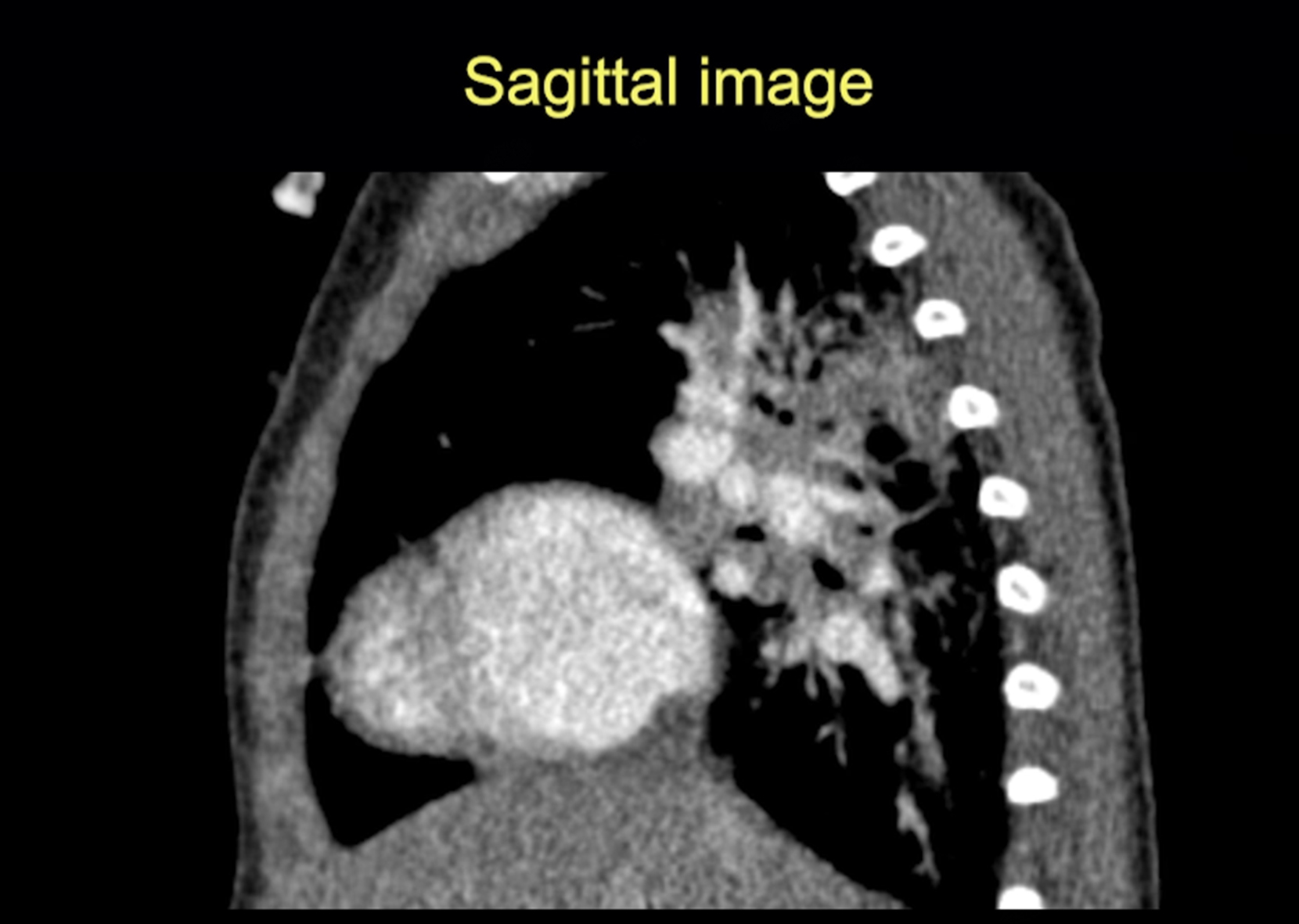

Supplement: Video 2 — Postoperative computed tomography imaging and echocardiogram. Video available at: https://www.jtcvs.org/article/S2666-2507(24)00167-6/fulltext. [file fx3.jpg]
